# Connecticut Veterinary Medical Association

**Published:** 1896-01

**Authors:** H. W. Eliot

**Affiliations:** Secretary


### CONNECTICUT VETERINARY MEDICAL ASSOCIATION.

THE regular meeting was held at the office of Dr. James Kelly, in New Haven, on the evening of December 3, 1895. The following answered to roll-call: Dr. Bland, of Waterbury, President; Drs. Ross, Kelly, Whitney, Lynch, and Isaacs, of New Haven; Prophett, of Bridgeport; Stoors, of Willimantic; Beckley, of Meriden; and Eliot, of Ansonia.

After the reading of the minutes of the last meeting a communication was read from Dr. Gardner, of Hartford, stating that he would be present at the next meeting and give the members a *résumé* of his labors with tuberculosis during the past year, and comprising statistics from a large number of herds, not only in this State, but also in Massachusetts and New York.

After the routine business was finished and in the absence of Dr. Potter, who was to read a paper on "Horseshoeing," Dr. Bland gave a detailed account of the operation of arytenectomy that he and Dr. Harger, of Philadelphia, performed on a horse in Waterbury.

Dr. Storrs told of several operations of cuneotomy that he had performed. He stated that 50 per cent. of his cases made good recoveries. Dr. Ross stated that about 75 per cent. of his operations for the same trouble were satisfactory.

After the meeting adjourned supper was served at Henkline's.

H. W. ELIOT,
Secretary.

